# Rare Cases of Syphilis

**Published:** 1881-08

**Authors:** Joseph Ransohoff

**Affiliations:** Professor of Anatomy and Clinical Surgery, Medical College of Ohio


### RARE CASES OF SYPHILIS.

By JOSEPH RANSOHOFF, M.D., F.R.C.S.,
Professor of Anatomy and Clinical Surgery, Medical College of Ohio.

The omission of an impure intercourse, the presence of the initial sclerosis, of mucous patches on the lips, of a cutaneous eruption and loss of hair render the recognition of syphilis in its earlier stages a task to which every tyro in medicine soon becomes equal. Aided or not by treatment, the evidences of disease successively disappear in the course of from one to five years, and in the majority of indivduals a restitution to a condition of perfect health is accomplished. Happily it is among the clinical rarities that we find palpable relapses of the disease after a period of quiescence extending from ten to twenty or more years. When, however, syphilis does produce these late manifestations, numerous factors conspire to make their recognition the test of diagnostic skill, and even annul at times, the possibilities of differentiation from the mere physical appearances and previous history. Regarding the latter, it should be remembered that without intentional distortion on the part of the patient, this is often incomplete from the fact that earlier constitutional manifestations did not exist, or their existence has been ignored or forgotten. In married women the absence of early muco cutaneous lesions is particularly frequent, the first intimation of a leutic taint being obtained from the existence of a greater or less number of characteristic destructive lesions of the skin.

The fact that adds most to the difficulty of always recognizing the late lesions of syphilis, is the absence of a distinctive structural element in the formation of the so-called gummatous tumor. Notwithstanding the indefatigable researches of Robin, Lancereaux, Virchow and Lebert, no characteristic elements or arrangement of them has been discovered in the gumma which would justify the use of the term "syphilom" suggested by E. Wagner and Virchow is now inclined to rank it with tumors of the connective-tissue type, recognizing in it a resemblance to the small-celled sarcoma. The most striking feature of the gumma is clinical in nature and depends on the extraordinary proclivity to rapid, fatty or caseous degeneration of the cells forming the growth, as a sequence of which its absorption or destructive ulceration will respectively occur. Another important clinical feature of the gumma pertains to the manner of its growth, the tendency being strong to enlarge by a process of agglomeration; nodules of varying size are developed in close approximation, confluence ensues and a chain of irregular tumor results, which undergoes retrograde changes first in the parts of oldest formation. It is in this manner that the serpiginous ulcerations of the skin and the peculiar appearances of specific lesions of the nerve centres are to be accounted for.

In the great preponderance of instances the previous history elucidates the nature of suspicious neoplasms, and their elasticity to the touch, their appearance in favorite situations, multiplicity and rapid disappearance under anti-specific treatment stamp them as syphilitic in origin. It is only when it appears singly and in places not usually its habitat that the late lesion in syphilis fails to be recognized, and engenders pardonable errors in diagnosis. Syphilitic tumors of muscles, mammary or lymphatic glands, and especially of the tongue, are particularly liable to be viewed as non-specific in nature and consequently subjected to at least irrational if not detrimental treatment.

Within the last year I have had an opportunity of witnessing three rather obscure cases of syphilis which presented sufficient interest to justify a detailed report.

Case I.—Æt. 42, civil engineer. Saw the patient in consultation with a distinguished clinician of Cincinnati. Rigid examination failed to reveal syphilitic antecedents. About two years before the consultation, the patient observed a uniform enlargement of some of the lymphatic glands of the neck and groin. After several months of growth, disintegration of some of the tumors gave origin to unhealthy ulcers yielding a yellowish discharge, moderate in quantity. During the last six months a dyspnœa supervened of sufficient severity to confine the patient to his bed. The physical examination of the patient revealed the presence of two firm yet elastic tumors of the size of an orange, on the right side of the neck behind the sterno-mastoid muscle. Without causing spontaneous pain they were quite sensitive to the touch. Their cutaneous covering presented no discoloration. On the same side, near the origin of the muscle mentioned, adhesions to the skin had supervened and were followed by its destruction and discharge of the character already described. In both groins the lymphatic glands presented uniform enlargements, tumors varying in size from a walnut to a peach being present. On the right side compression of the femoral vein had produced very marked œdema of the extremity. Physical exploration of the lungs and heart failing to elicit lesions accountable for the dyspnœa, this was attributed to a compression of the trachea and bronchi by enlarged bronchial glands. The diagnosis of multiple lymphadenoma seemed the proper one, and the pallor of the skin and mucous membrane rendered microscopic examination of the blood superfluous. A view of the left leg, accidentally obtained, displayed several round cicatrices in close proximity to each other, and aroused the first suspicion of the possible existence of tertiary syphilis of the lymphatics. Mercurial inunctions were carefully practiced in conjunction with the administration of the iodide of potassium. In six weeks the superficial tumors and dyspnœa had disappeared, and the patient was sufficiently restored to venture on a trip to the country for recreation. Unfortunately, through neglect on the part of the patient, an untreated relapse of the difficulty caused his death after a respite of six months. The autopsy revealed an enlargement of the bronchial and mesenteric glands and gummatous tumors in the right lobe of the liver. Specimens for microscopic examination could not be obtained.

According to Lancereaux, tertiary enlargements of the deep-seated lymphatic glands are among the most common of syphilitic lesions, and bear to the gummata of the viscera the same relation that do the superficial ganglia to the affections of the muco-cutaneous structures in the earlier periods of the disease. This opinion of the eminent syphilologist does not correspond with general experience, and tertiary syphilitic adenopathy must be considered a rather rare affection. Indeed, the very absence of ganglionic enlargement at times becomes the distinguishing feature of syphilitic lesions. Destructive ulcerations of the septum nasi, palate, tongue and epiglottis are often encountered without engorgement of the lymphatic glands in the vicinity, while, as in the case narrated, syphilitic infiltration and subsequent degeneration may befall them without appreciable change in the structures whence their lymph supply is obtained. This corresponds to what so frequently appears in the earlier stages of syphilis in which the epitrochlear and cervical glands are enlarged before the appearance of cutaneous manifestations on arm and face.

Case II.—Mrs. B , æt. 47, mother of seven children, of which two died in infancy. History of three miscarriages. Claims to have been healthy until two years before her appearance at the Dispensary of Medical College of Ohio. At that time a swelling appeared in each breast which was supposed to be of a cancerous nature, and on account of which both breasts were amputated by an advertising specialist of Cincinnati, one year before her appearance at the College clinic. Relief was now sought for the relapse of the supposed tumor. In the place of mammary glands and nipples are found two cicatrices elongated and parallel to the body-axis. The cicatrices are somewhat indurated; that on the right side bearing upon its surface a serpiginous ulceration two inches in length with sharply defined edges and yellowish floor. The axillary glands are not involved. Further investigations revealed the presence of round and oval ulcers on the left thigh, right arm and shoulder. Although at the first examination the existence of previous syphilitic manifestation was strenuously denied, it was subsequently determined that the patient's husband had been afflicted with the disease before marriage, and was even now suffering from a tubercular syphilide.

The diagnosis of syphilis having been substantiated, thirty grain doses of the iodide of potassium were administered three times a day conjointly with the extract of malt. An ethereal solution of iodoform was applied to the ulcers. In four weeks the patient was discharged in a condition of good health, and no relapse has since recurred.

Aside from its interest as a clinical rarity this case illustrates only too well the baneful effects of the knife when needlessly guided by an ignorant hand. Had a careful study of them been made, the gummata of the breast could not have been mistaken for carcinomatous growths, and the disfiguring operations would have been dispensed with. Fortunately tertiary lesions of the breast are among the most unusual of syphilitic changes, since no reference to them appears in even the comprehensive textbooks of native and foreign writers. The literature of these cases is not very abundant, but in every instance of which the record was accessible, I found that the mammary growth was not the only indication of the syphilitic diathesis. Two years ago Cheever reported the case of a negro with syphilitic enlargement of the left breast also attended with specific growths in other parts of the body. On this account an operation was deferred and iodides administered. In three weeks the mammary tumor had quite disappeared.

Among the most deceptive of syphilitic lesions, although described by most syphilologists, are gummata of the muscles and inter-muscular connective tissue. A large proportion of the cases encountered affect the tongue, patients being actuated to seek relief by the dread of cancer and the inconvenience and suffering endured.

Even without the confession of a luetic taint, gummata of this organ are as a rule readily recognized, appearing as round or oval and more or less indurated growths in the substance of the tongue or immediately underneath the mucous membrane of the dorsum, which from the pressure exerted upon it from below, presents a bright red, glistening appearance, and is elevated or not according to the depth of the infiltration. Gummata usually develop rapidly, and when softening ensues give rise to non-ichorous discharges. The appearance of a suspicious growth on the under surface of the tongue militates against its specific nature, since, according to Fournier, gummata never appear in this region. The multiplicity of the abnormal deposits is likewise evidence of their specific character, while induration *per se* is neither characteristic of syphilis nor epithelioma. It is estimated by Agnew in his recent work that, as a diagnostic feature of syphilis of the tongue the induration follows the ulceration in a manner directly opposed to the progress of epithelial tumors. This view is the more incomprehensible, for in both instances induration must of necessity precede the ulceration, since the latter is but the disintregation of the infiltrated tissues. Cases, however, unquestionably occur in which a differential diagnosis becomes impossible, if for no other reason than that the prolonged irritation of the tongue may give rise to a carcinomatous growth at or near the side of a gumma. Quite recently, Langenbeck has recorded a case of this kind in which anti-specific therapy relieved the syphilitic lesions of the tongue without affecting the malignant ulcer on the surface of the organ or the accompanying enlargements of the cervical glands.

Case III —One year ago I was called to relieve the sufferings of a Mrs. L., whose family physician was absent from the city. The patient, aet. 60, was tortured by lancinating pains in the tongue. Inspection revealed a large irregular ulcer on the dorsum of the organ, extending to and involving the right margin, with raised border, yellow and ragged floor, sparingly secreting a watery fluid of greenish hue. Functional disturbances were marked; mastication and deglutition painful, and speech indistinct. Right submaxillary gland although enlarged was not very much indurated. Without entering into the previous history of the case, a conviction of the malignant nature of the ulceration forced itself upon me, and to relieve the suffering an anodyne was administered with temporary success. Subsequent information of the patient's antecedents, however, led to the suspicion of a latent syphilis. Anti-specific treatment with inunctions was substituted in consequence, and complete recovery ensued.

This case seemed of sufficient importance to merit presentation to your learned body, since all the local symptoms indicated the presence of a malignant growth and the correction of the first erroneous impression saved the patient from one of the gravest of surgical operations. The lancinating pain, the involvment of the cervical lymphatics, indurated borders of the ulcer and age of the patient are all regarded as indicative of carcinoma, but to rely upon them alone opens a pathway to fatal errors. In 1872 a physician presented himself to Langenbeck with a supposed cancer of the tongue, with an operation in view. Thirty years before he had suffered from primary and secondary syphilis, since which time the poison had given no evidence of its being. Under scientific treatment the lingual disease vanished. Cases like the one I have taken the liberty to submit have been but sparingly recorded. In 1877 Vinzenso Zanini published the history of a patient in whom all the symptoms indicative of carcinoma existed, but who recovered rapidly under the ordinary anti-syphilitic treatment. Much as it is to be regretted, it must be conceded that in certain rare cases no single factor or combination of them enables a distinction to be made between gummatous tumors or ulcers and epithilial growths of the tongue. The possible transformation of the one into the other but adds to the difficulty of a problem, the solution of which is at times made apparent only by anti-syphilitic therapy cautiously administered. It is important to remember that in two of the above cases the subjects of observation were married women, advanced in years. In both syphilis was acquired without the knowledge of the patient, and that without early recognized manifestations.

